# Dental biofilm serves as an ecological reservoir of acidogenic pathobionts in head and neck cancer patients with radiotherapy-related caries

**DOI:** 10.1128/msphere.00257-25

**Published:** 2025-06-30

**Authors:** Julia S. Bruno, Vitor Heidrich, Felipe C. F. Restini, Tatiana M. M. T. Alves, Wanessa Miranda-Silva, Franciele H. Knebel, Elisangela M. Cóser, Lilian T. Inoue, Paula F. Asprino, Anamaria A. Camargo, Eduardo R. Fregnani

**Affiliations:** 1 Molecular Oncology Center, Hospital Sírio-Libanês 42522, São Paulo, Brazil; 2 CIBIO, University of Trento 19034, Trento, Italy; 3 Radiotherapy Department, Hospital Sírio-Libanês 42522, São Paulo, Brazil; University of California Davis, Davis, California, USA

**Keywords:** oral microbiome, radiotherapy, toxicity, biofilm, dental caries, supportive care

## Abstract

Radiotherapy-related caries (RRC) is an aggressive and debilitating oral toxicity that affects half of the patients who undergo radiotherapy for head and neck cancer. However, the etiology of RRC is not fully established, and there are no clinically validated methods for preventing it. To gain a better understanding of the risk factors and the microbiome’s role in causing RRC, we compared clinicopathological characteristics, oncological treatment regimens, oral health condition, and the oral microbiota at three different oral sites of radiotherapy-treated patients with (RRC+) and without radiotherapy-related caries (RRC−). We observed no significant differences between these groups in the clinicopathological characteristics and treatment regimens. However, RRC+ patients were older and had poorer oral health conditions at the start of the radiotherapy treatment, with a lower number of teeth and a higher proportion of rehabilitated teeth. RRC+ patients had lower microbiota diversity and the dental biofilm of RRC+ patients displayed striking alterations in microbiome composition compared to RRC− patients, including enrichment of acidogenic species and altered metabolic potential, with a higher abundance of genes linked to energy-related pathways associated with the synthesis of amino acids and sugars. We also compared the microbiota of RRC+ tissue with conventional caries tissue, revealing lower bacterial diversity and enrichment of Lactobacillaceae members in RRC+. The insights into the irradiated oral microbiota enhance the understanding of RRC etiology and highlight the potential for microbial-targeted therapies in its prevention and treatment.

**IMPORTANCE:**

This study focuses on a dedicated collection of diverse oral sites to comprehensively investigate microbial differences between patients who develop RRC and those who do not. RRC is a severe oral disease that profoundly impacts on the oral health and overall quality of life of cancer survivors. Leveraging shotgun metagenomics, we characterize the unique microbial variations in *in vivo* irradiated dental biofilms, unveiling novel insights into the microbial ecology of radiotherapy-treated patients. Furthermore, this research integrates extensive data on oral health and oncological profiles, providing a comprehensive understanding of the intricate relationship between oral microbial communities and the outcomes of radiotherapy-induced toxicity.

## INTRODUCTION

Head and neck cancer (HNC) has a high worldwide incidence and mortality rate (1). Around 75% of HNC patients undergo radiotherapy as primary or adjuvant treatment (2). While advanced treatments (i.e., proton therapy) show promise in preserving healthy tissues, intensity-modulated therapy remains the gold standard for HNC (1, 2). Post-radiotherapy toxicities are frequent and can significantly impact the short- and long-term patient’s quality of life. The most predominant acute oral toxicities are mucositis, opportunistic infections, and neurosensory disorders (3, 4). In addition, patients are chronically at risk of experiencing tissue fibrosis, osteoradionecrosis, salivary gland dysfunction, and radiation-related caries (RRC) (5, 6).

RRC is a biofilm-dependent pathology that affects 29%–57% of HNC patients who underwent radiotherapy (7) and appears within the first 3 months. It is considered harmful to the stomatognathic system due to the fast clinical progression and the limited effectiveness of preventive measures (5). Unlike conventional caries (CC), RRC occurs in specific areas of the teeth (cervical portion and crown cusps) and often has a painless progression (8). In later stages, RRC appears as enamel decalcification lines and brownish dentin. The most severe cases result in coronary amputations (8). In addition to damaging dental tissue, RRC increases the risk of developing osteoradionecrosis in the jaws (9, 10).

RRC etiology still needs to be fully understood. No specific risk factors have been identified, but factors related to traditional tooth decay, such as diet and oral hygiene habits, may also contribute to RRC (10, 11). A lower number of teeth before radiotherapy (10), gingival recession post-treatment (12), and poor oral hygiene (10) have been reported to increase the risk of RRC. Additionally, factors associated with cancer treatment, such as the direct impact of radiation on dental tissues (13–16), and oral microbiome dysbiosis, may also play a role in the development of RRC (17–22). Although most of these studies report a decrease in oral bacteria diversity after radiotherapy (17–19), there are no studies comparing the oral microbiota of irradiated patients with the RRC tissue. And no specific microbial species or signature has been linked to RRC.

RRC is a chronic condition that significantly impacts a person’s quality of life and daily routine (23, 24). Regrettably, there are currently no clinically validated methods for preventing it and restorative management of RRC can be challenging. However, utilizing precise molecular techniques to comprehensively understand the oral microbiome and identify risk factors and specific targets may help develop effective preventive strategies for managing biofilm-related oral pathologies such as RRC (25, 26). Here, we compare clinicopathological characteristics, oncological treatment regimens and toxicities, oral health condition, and oral microbiota diversity and composition of the oral mucosa, dental biofilm, and gingival crevicular fluid of radiotherapy-treated HNC patients with (RRC+) and without RRC (RRC−). We also compared RRC tissue with carious tissue from healthy subjects with CC. Through an extensive metagenomic characterization of irradiated dental biofilm and carious tissue, our data provide insights into RRC etiology that might contribute to the development of microbial-targeted therapies to prevent RRC and restore oral health in HNC survivors.

## MATERIALS AND METHODS

### Study population and clinical features

We enrolled 33 HNC patients treated with radiotherapy at Hospital Sírio-Libanês, São Paulo, Brazil. All radiotherapy-treated patients received dental evaluations and specialized oral care following established guidelines (5). These patients were divided into a group that developed (RRC+, *n* = 17) and another group that did not develop RRC (RRC−, *n* = 16). We also recruited volunteers with no history of cancer with conventional caries (CC, *n* = 16). This cross-sectional study was approved by the Research Ethics Committee of Hospital Sírio-Libanês (#HSL2018-71). All the patients signed the informed written consent prior to the study and under the Declaration of Helsinki. The groups did not differ in sex or tobacco consumption, but patients in the RRC+ group were significantly older, with mean age of 68.5 ± 10.5 years, compared to 54.9 ± 11.5 years for the RRC− group, and 56.8 ± 18.4 years for the CC group (*P*-value = 0.014, one-way ANOVA, Table S1).

Most of the HNC patients had squamous cell carcinoma in the oral cavity and were treated with volumetric-modulated arc therapy. There were no significant differences in the clinicopathological characteristics and treatment regimen between the two groups of HNC patients (Table 1) (Table S2). Noteworthy, there was no difference in the radiation dose on the major salivary glands and in the incidence of xerostomia between patients with or without RRC (Table 1). However, RRC+ patients had poorer oral health conditions at the start of the radiotherapy treatment, with a lower number of teeth and a higher proportion of rehabilitated teeth compared to RCC− patients, indicating that poor oral health is a major risk factor for RRC development (Table 1).

**TABLE 1:** Radiotherapy data and oral condition status of the study population^*b*^^,^^*c*^

	Study population		*P*-value, test
	RRC− (*n* = 16)	RRC+ (*n* = 17)	
Radiotherapy—no. (%)			
V-MAT	14 (87.5%)	12 (70.58%)	0.4823, c
3D-Conformal	1 (6.25%)	3 (17.64%)	
Step-and-shoot	1 (6.25%)	2 (11.76%)	
Organs at risk—in cGy (median, Q1–Q3)			
Ipsilateral parotid gland	2,550, 2,355–3,531	2,529, 1,763–3,934	0.6833, f
Contralateral parotid gland	1,565, 930–2,027	2,326, 1,103, 2,529	0.2298, g
Ipsilateral submandibular gland	6,321, 4,596–6,754	6,088, 5,537–6,457	0.4559, f
Contralateral submandibular gland	3,254, 1,594–5,101	4,863, 2,621–5,833	0.3666, f
Oral cavity	4,526, 3,334–4,891	3,745, 1,950–5,003	0.4613, g
Dental arches	3,074, 2,318–4,243	2,759, 1,911–3,230	0.3245, g
Gross tumor volume	6,600, 6,000–6,996	6,798, 6,450–6,996	0.2864, f
Oral condition—before treatment			
Number of teeth (average, min–max)	26.13, 15–32	17.24, 1–28	**0.0009**, g^*d*^
Number of implants (average, min–max)	0.81, 0–5	1.41, 0–7	0.7937, f
Rehabilitated teeth^*a*^ (median in %, Q1–Q3)	39, 22.6–59.7	64.7, 45.5–80.9	**0.0272**, g
Re^*d*^sidual root^*a*^ (median in %, Q1–Q3)	0.0–0	0.0–2.5	0.1026, f

^*a*^ Total number of affected teeth/total number of teeth in the oral cavity.

^*b*^ cGy: centi-gray.

^*c*^ Q1: first quartile; Q3: third quartile; Tests: c: Fisher-Freeman-Halton Test; f: Mann-Whitney Test; g: Unpaired *t*-test.

^*d*^ Bold values indicate statistically significant *P*-values.

### Sample collection

Oral mucosa, dental biofilm, gingival crevicular fluid, and demineralized dental tissue (RRC and CC) samples were collected simultaneously on the day of the RRC/CC diagnosis. Irradiated patients (RRC− and RRC+ groups) were time-matched based on the number of months post-radiotherapy, with a median follow-up of 40.5 months (Fig. S1), thereby standardizing the temporal window for the potential onset and detection of RRC across all participants.

The samples were collected per site: Oral mucosa: rubbing the buccal mucosa, alveolar sulcus, and tongue dorsum with a sterile swab (Inlab, São Paulo, Brazil); Dental biofilm: rubbing the vestibular supragingival tooth of the upper and lower arches with the sterile swab (Inlab, São Paulo, Brazil); Gingival crevicular fluid: absorption with 12 paper points units (F3 Cellpack Protaper) (Dentsply Maillefer, Ballaigues, Switzerland) in the gingival sulcus for 60 s (only patients with no presence of active periodontal disease or bleeding on probing); Carious tissue (CC and RRC): the demineralized carious tissue was collected with a sterile Lucas curette instrument.

### Clinical data collection and analysis

The computed tomography scans were uploaded in the Eclipse External Beam Planning software (version 15.6, Varian Medical Systems, Inc, Palo Alto, USA) for delineating the primary tumor, organs at risk (parotid salivary glands, submandibular glands, mandibular jaw), dental arches, and oral cavities. The planning target volume was created in accordance with the Radiation Therapy Oncology Group (RTOG) reports. Dose calculation was performed with an anisotropic analytical algorithm (AAA_10028), grid resolution of 0.2 cm, and heterogeneity correction. Oral mucositis was measured during radiotherapy based on the World Health Organization (WHO) grading scale (27). Xerostomia was measured during sample collection based on the Common Terminology Criteria for Adverse Events (CTCAE) scale (version 3.0). Oral condition data were collected based on oral examination, panoramic radiograph, or computed tomography scan. The number of teeth was measured by counting natural and rehabilitated functional elements. The number of implants was counted only in rehabilitated implants. Rehabilitated teeth were counted according to the previous history of dental procedure (e.g., composite resin). Residual roots were accounted by tooth roots remaining above or below the bone crest.

Differences in demographic, dental, and oncological variables were analyzed according to the characteristics of numerical and binary variables. Binary variables were analyzed through contingency tables using the Chi-square, Fisher’s exact, or Fisher-Freeman-Halton tests. Numerical variables were tested for normality with the Shapiro-Wilk test and underwent the appropriate test. The non-parametric data were analyzed by the Mann-Whitney *U* or Kruskal-Wallis tests, and the parametric data were analyzed by the one-way ANOVA test. The use of each test is indicated in the corresponding table. The *p-value* and *q-value* were considered significant when <0.05.

### DNA extraction

For oral mucosa, dental biofilm, and gingival crevicular fluid samples, 600 µL of Tris-EDTA buffer (10 mM Tris, 1 mM EDTA, pH 8.0) was added to the Eppendorf to transfer the biological material to the buffer. PureLinkTM RNase A (20 mg/mL, Thermo Fisher Scientific, Waltham, USA) was added (6 µL for oral mucosa and dental biofilm samples and 8 µL for gingival crevicular fluid samples), followed by 30 µL of Protease 7.5AU (QIAGEN, Hilden, Germany). The DNA was extracted using the QIAamp DNA Blood&Tissue Kit (QIAGEN, Hilden, Germany) according to the manufacturer’s protocol.

For carious tissue (CC and RRC samples), the pre-processing step consisted of adding 900 µL of TES buffer (Tris HCl pH8.0 1 mM + EDTA pH8.0 + SDS 10%) and 50 µL of Proteinase K 600 mAU/mL (QIAGEN, Hilden, Germany) for overnight incubation. The processing step consisted of adding 2 µL of PureLink RNaseA (20 mg/mL, Thermo Fisher Scientific, Waltham, USA) and 1 mL of UltraPure Buffer-Saturated Phenol (Thermo Fisher Scientific, Waltham, USA) for phase separation. The supernatant was collected after up-and-down movement with (i) phenol:chloroform:isoamyl alcohol (25:24:1), (ii) chloroform:isoamyl alcohol (24:1), and (iii) isopropanol and 3 M sodium acetate, respectively. The pellet was cleaned twice with ice-cold 70% ethanol and resuspended in Tris-EDTA buffer (10 mM Tris, 1 mM EDTA, pH8.0).

### 16S rRNA gene amplification and sequencing and data analysis

Amplification was performed with the KAPA HiFi HotStart PCR Kit (Kapa Biosystem, Cape Town, South Africa) and the use of specific primers for the V3-V4 regions of the 16S rRNA gene (F (5′-TCGTCGGCAGCGTCAGATGTGTATAAGAGACAGCCTACGGG-3′) and R (5′-GTCTCGTGGGCTCGGAGATGTGTATAAGAGACAGGACTAC-3′)). The purification of the PCR products was performed with the AMPure XP PCR Purification Kit (Beckman Coulter, Brea, USA), and the samples were quantified by fluorimetry with the Qubit dsDNA HS Assay (Thermo Fisher Scientific, Waltham, USA). The pool was quantified with the kit NEBNext DNA Library Prep Master Mix Set for Illumina (New England Biolabs, Ipswich, USA), and the protocol of the kit MiSeq Reagent Kit V3 (Illumina, San Diego, USA) was performed to sequence the library on the Illumina MiSeq platform (Illumina, San Diego, USA).

The amplicon-sequencing data were pre-processed in the QIIME2 framework, using the DADA2 pipeline to generate amplicon sequence variants (ASVs). ASVs were taxonomically assigned using the HOMD database (v15.23) (28) and the VSEARCH tool. Finally, the data were normalized by Scaling with Ranked Subsampling at 13,385 reads/sample. Alpha diversity (29) was calculated using the Shannon index (30) and the observed number of ASVs (as a proxy for richness). The beta diversity was measured by the Bray-Curtis dissimilarity (31) index and visualized by PCoA. The overall compositional difference between the groups was assessed by the PERMANOVA test. Differential abundance of taxa between groups was assessed through the ANCOM-BC method (32, 33). To evaluate the association between irradiated dose in dental arches and salivary glands and microbial features, the R package MaAsLin2 (34) was used.

### Dental biofilm shotgun metagenomic sequencing and data analysis

For each dental biofilm sample, the DNA was re-quantified by fluorimetry with the Qubit dsDNA HS Assay (Thermo Fisher Scientific, Waltham, USA) and diluted to 1 ng. The DNA tagmentation, PCR, and libraries were prepared using the Nextera-XT DNA Library Prep Kit (Illumina, San Diego, USA) following the steps of the manufacturer’s protocol. The library clean-up was performed with the AMPure XP PCR Purification (Beckman Coulter, Brea, USA). The libraries were sequenced on the Illumina NovaSeq 6000 platform (Illumina, San Diego, USA).

The shotgun metagenomic sequencing data were pre-processed using kneaddata with default parameters (https://github.com/biobakery/kneaddata) for the removal of low-quality and contaminating reads. Low-quality reads are the ones shorter than 75 bp after trimming poor-quality ends (average quality <20 for a sliding window of 4 bp) with Trimmomatic (35). Contaminating reads are identified by mapping the remaining reads with BowTie2 (36) against the reference human genome (hg19) and the bacteriophage phiX174DNA (Illumina spike-in). MetaPhlAn4 (37) was used to profile taxonomic compositions with the vJun23_202307 database. HUMAnN3 was used to analyze the functional profile by quantification of the metabolic pathways per species with the MetaCyc database (38).

## RESULTS

### Samples collected and sequencing output

We collected a total of 132 samples, divided by group and sampling site as follows: the RRC+ group (*n* = 17) included 17 samples from each site (oral mucosa, dental biofilm, gingival crevicular fluid, and RRC tissue), totaling 68 samples. The RRC− group (*n* = 16) included 16 samples from each of the three sites (oral mucosa, dental biofilm, and gingival crevicular fluid), totaling 48 samples. The CC group (*n* = 16) contributed with 16 samples of CC tissue. Of the 132 collected samples, 3 failed to amplify during library construction (1 from RRC tissue and 2 from the CC tissue) and were, therefore, not subjected to sequencing.

16S rRNA gene sequencing was performed on 129 samples. Considering all samples together, 30,969,617 raw reads were generated (range: 24,681–565,686), with an average of 150,332 raw reads/sample. After DADA2, an average of 103,642 high-quality reads (64.8% of the raw reads) were obtained. We established a sequencing depth cutoff of 3,000 reads (39), and no sample was excluded for further analysis.

Shotgun metagenomic sequencing was performed on a subset of the dental biofilm samples of the RRC+ (12 samples) and RRC− groups (12 samples). After kneaddata, an average of 16.9M pre-processed reads were obtained per sample (range: 1.3–79.8 M pre-processed reads), with sample-wise pre-processing statistics available in Data S1.

### Microbiota diversity and composition of radiation-related caries

**Fig 1:**
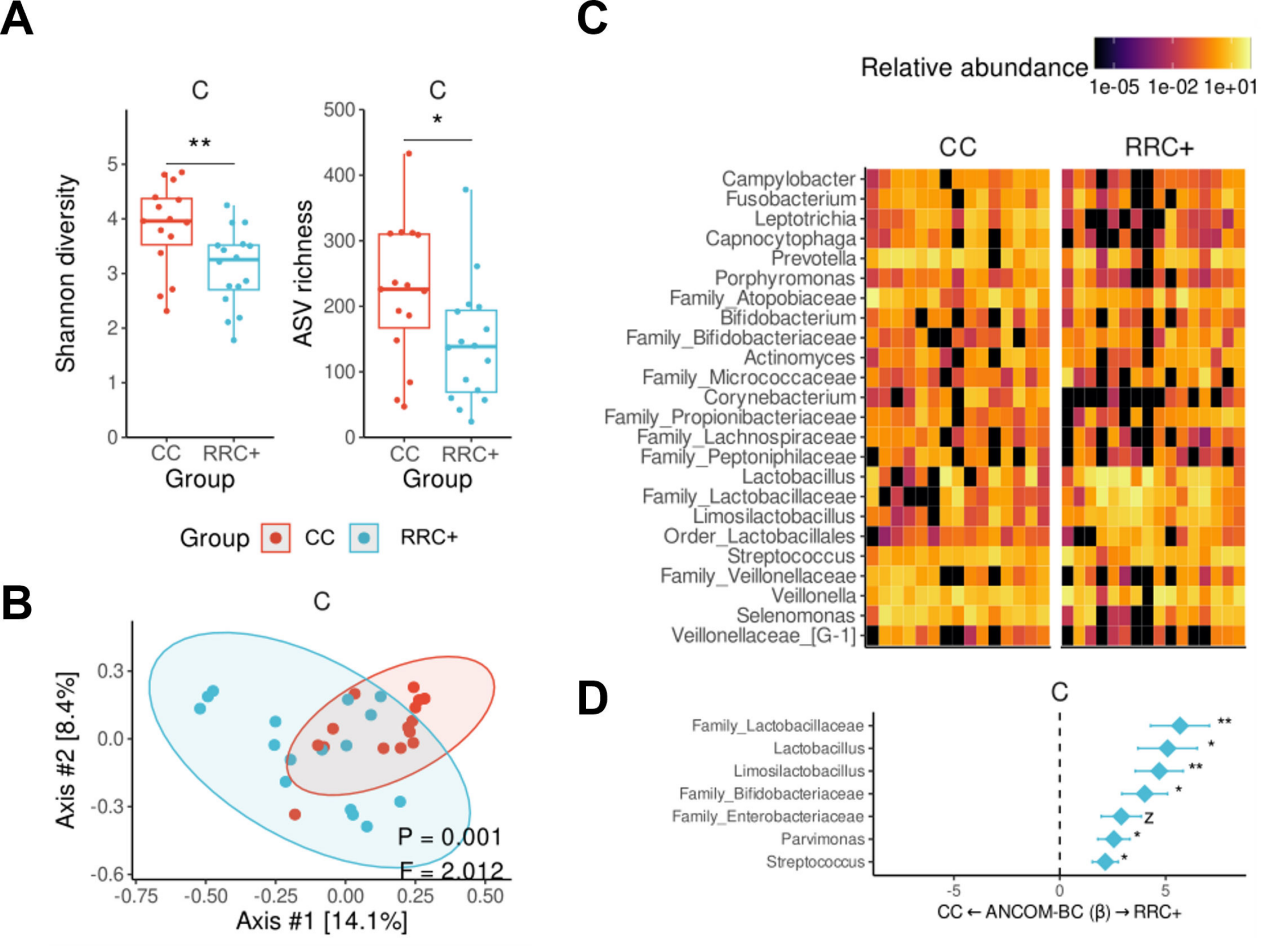
Microbiota in radiation-related caries show lower diversity and a predominance of Lactobacillaceae members. Alpha diversity analysis is measured by the Shannon index (**A**) and beta diversity measured by the Bray-Curtis dissimilarity metric (**B**). The relative abundance of 25 bacterial genera (selection criteria: at least 1% relative abundance in ≥25% of samples or ≥30% relative abundance in at least one sample) elucidates the difference between the overall composition between RRC and CC (**C**) with significant differences assessed with ANCOM-BC (**D**). Each box represents the median and percentiles (25th and 75th), with lines extending to the extreme (at most 1.5 times the size of the box).

We performed 16S rRNA microbiota profiling of RRC and carious tissue from patients with no previous history of cancer or radiotherapy and with CC. RRC+ had lower bacterial diversity (Shannon’s index) and lower richness of observed ASVs compared to CC (Fig. 1A, Mann-Whitney *U* test, *P*-value = 0.009 and *P*-value = 0.033, respectively). The bacterial composition also differed between RRC+ and CC (Fig. 1B *P*-value = 0.001, *F* = 2.012, PERMANOVA), with RRC tissue displaying a more variable composition as observed by principal coordinate analysis (PCoA). The overall genus-level microbiota compositions of the carious tissue of each patient are shown in Fig. 1C, highlighting an evident predominance of Lactobacillaceae members among RRC+, including *Lactobacillus, Limosilactobacillus*, and an unidentified genus of the family, which together often composed >50% of the carious tissue microbiota in this group (38% prevalence vs 7% in CC). To more rigorously evaluate those differences, we ran a differential abundance analysis with ANCOM-BC, revealing the aforementioned genera were all significantly enriched in RRC+, along with other genera such as *Parvimonas* and *Streptococcus*. In contrast, no significant enrichment was observed in CC when compared to RRC+ (Fig. 1D).

### Oral microbiota diversity and composition in HNC patients treated with radiotherapy

**Fig 2:**
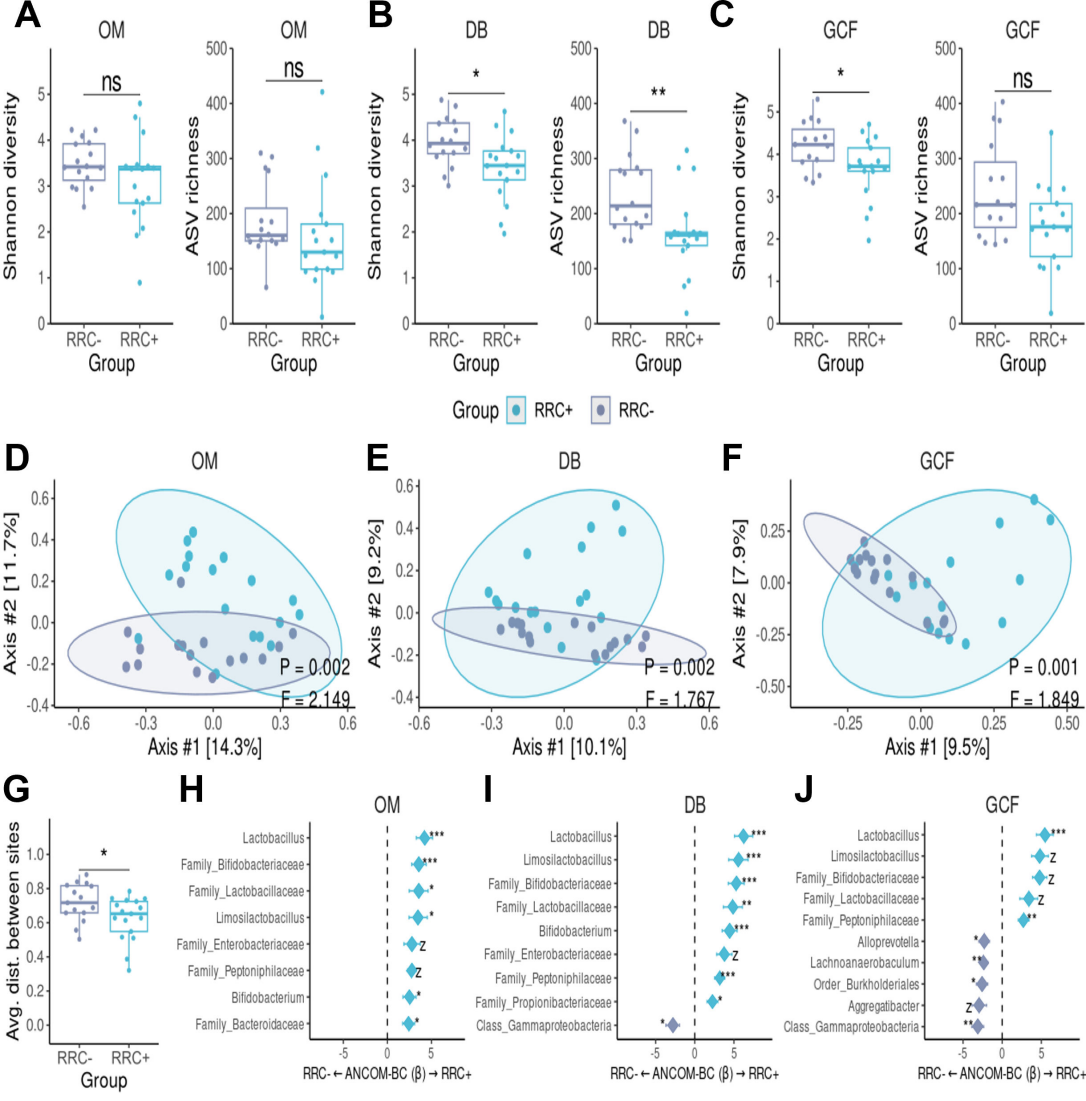
Oral sites of irradiated RRC-affected patients present lower diversity and more similar microbial composition. Oral mucosa (OM), dental biofilm (DB), and gingival crevicular fluid (GCF) are represented in (**A, D, H**), (**B, E, I**) and (**C, F, J**), respectively. Alpha diversity was measured with the Shannon Index (left) and ASV richness (right) (**A–C**). Beta diversity was calculated with the Bray-Curtis dissimilarity metric and represented by PCoA (**D**–**F**). The compositional distance between the oral sites of each group was compared by the Mann-Whitney *U* test (**G**). The compositional analysis difference was measured using ANCOM-BC at genus level with significantly enriched genera shown in (**H–J**). Each box represents the median and percentiles (25th and 75th), with lines extending to the extreme (at most 1.5 times the size of the box). The asterisk represents statistical significance: *, *P*-value < 0.05; **, *P*-value < 0.01; ***, *P*-value < 0.001; *z*, structural zero.

We next examined the diversity and composition of the oral microbiota in HNC patients with and without RRC at three distinct sites: oral mucosa, dental biofilm, and gingival crevicular fluid. Our findings revealed that RRC+ patients displayed lower bacterial diversity, as measured by the Shannon’s index and ASV richness, in the dental biofilm (Fig. 2B, Shannon’s index *P*-value = 0.017 and ASV richness *P*-value = 0.006, Mann-Whitney *U* test), and gingival crevicular fluid (Fig. 2C, Shanonn’s index *P*-value = 0.037 and ASV richness *P*-value = 0.069, Mann-Whitney *U* test), but not in the oral mucosa (Fig. 2A, Shannon’s index *P*-value = 0.160 and ASV richness *P*-value = 0.130, Mann-Whitney *U* test).

We also found that bacterial composition in RRC+ patients differed significantly from RRC− patients at all oral sites (Fig. 2D through F. Oral mucosa: *P*-value = 0.002, *F* = 2.149; dental biofilm: *P*-value = 0.002, *F* = 1.767; gingival crevicular fluid: *P*-value = 0.001, *F* = 1.849, PERMANOVA). Interestingly, the presence of RRC in the oral cavity led to increased similarity in the microbiota among the three oral sites, as evidenced by the decreased average intra-subject distance between oral sites in the RRC+ group (Fig. 2G *P*-value = 0.049, Mann-Whitney *U* test).

Genera such as *Lactobacillus*, *Bifidobacterium*, and *Limosilactobacillus* were enriched in the oral mucosa of RRC+ patients (Fig. 2H). The dental biofilm showed the most notable differences (*β* coefficient >5) in bacterial composition between the RRC+ and RRC− groups, with enrichment of *Lactobacillus*, *Bifidobacterium,* and *Limosilactobacillus* in RCC+ patients (Fig. 2I). In the gingival crevicular fluid, no compositional difference was found related to the main periodontopathogens. However, similar to the other sites, *Lactobacillu*s was enriched in RRC+ patients (Fig. 2J).

As the RRC incidence was previously associated with the irradiation dose in the parotid salivary gland (8), we used microbiota multivariable association with linear models (MaAsLin2) to associate the median or maximum dose irradiated in the parotid gland with the amplicon-based sequencing data from all investigated oral sites, but no significant associations were found (Data S2).

### Enrichment of acid-producer species in the dental biofilm of HNC patients with RRC

Overall, our findings suggest that the most prominent microbial shift occurred in dental biofilm. Biofilm dynamics play a key role in the physiopathology of RCC and CC (40, 41). Therefore, we next performed shotgun metagenomic sequencing on dental biofilm samples of RRC− and RRC+ patients to further characterize the irradiated dental biofilm at the species-level and identify potential metabolic pathways associated with RRC’s aggressive clinical behavior.

Metagenomic data confirmed that the dental biofilm of RRC+ patients have lower bacterial diversity compared to RRC− patients. This was indicated by a lower Shannon index (*P*-value = 0.012, Mann-Whitney *U* test) and a lower species-level genome bin richness (*P*-value = 0.01, Mann-Whitney *U* test) (Fig. S2a). Metagenomic data also confirmed the significant compositional differences between RRC+ and RRC− groups, as measured by the Bray-Curtis dissimilarity index (Fig. S2b *P*-value = 0.016, *F* = 1.77, PERMANOVA).

**Fig 3:**
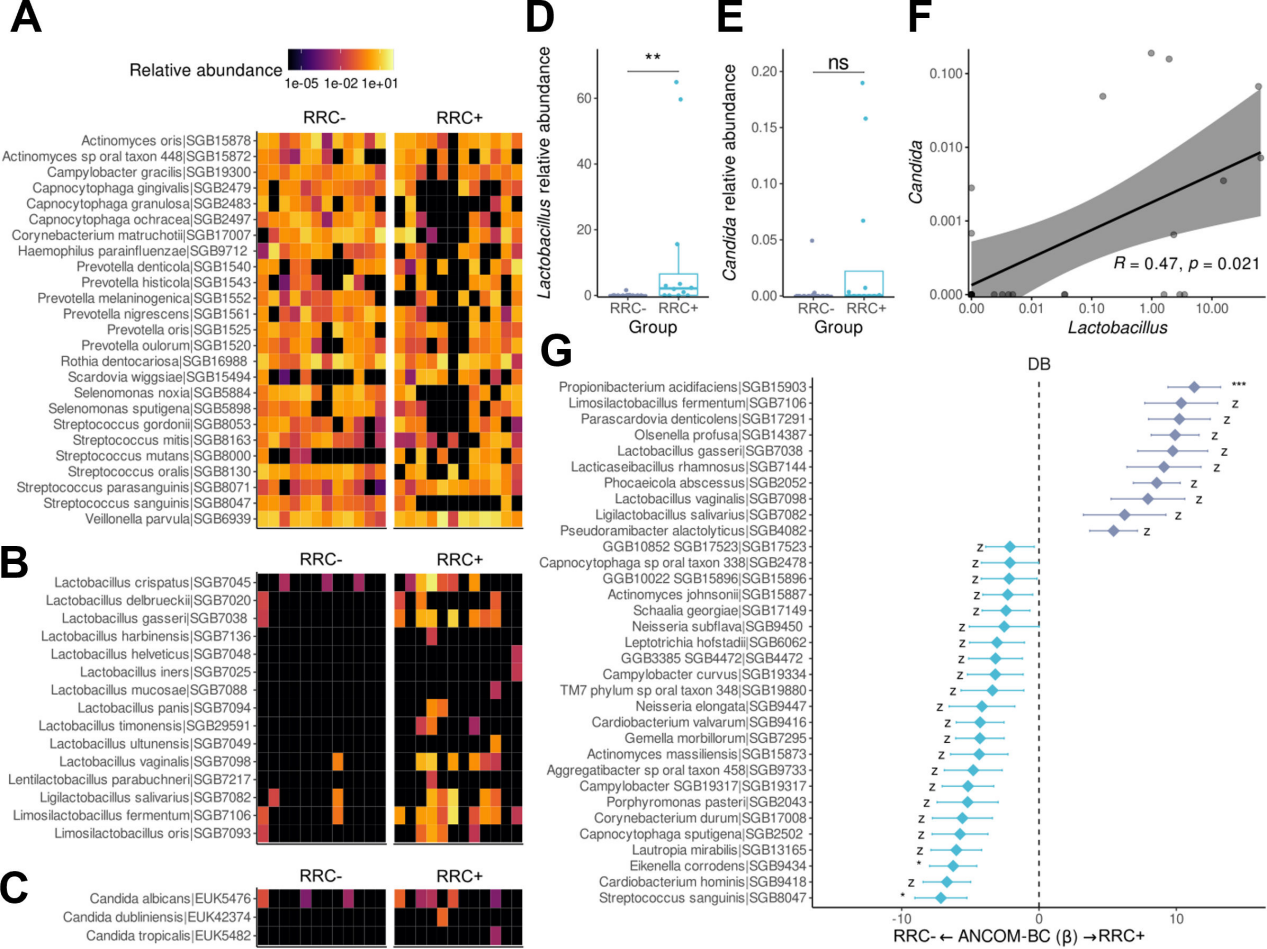
The lack of commensal species and enrichment of acid-producing bacteria characterize the dental biofilm of patients with RRC. Heatmap represents the relative abundance of the 25 species most abundant in all samples (**A**), lactic acid bacteria species (**B**), and *Candida* species (**C**). Relative abundance of *Lactobacillus* (**D**) and *Candida* (**E**) are represented by boxplots. The relative abundance correlation between *Lactobacillus* and *Candida* is represented at (**F**). The compositional analysis difference was measured using ANCOM-BC in the dental biofilm of the RRC+ group (**G**). Each box represents the median and percentiles (25th and 75th), with lines extending to the extreme (at most 1.5 times the size of the box). The asterisk represents statistical significance: *, *P*-value < 0.05; **, *P*-value < 0.01; ***, *P*-value < 0.001; *z*, structural zero. Note: Taxonomic nomenclature updated: *Bifidobacterium denticolens to Parascardovia denticolens* (42).

When visually analyzing the species-level relative abundance pattern between RRC+ and RRC− groups (Fig. 3A through C), we observed that the RRC+ group had a strikingly different profile, mainly due to the predominance of lactic acid bacteria species in the RRC+ group (Fig. 3B). Indeed, *L. harbinensis*, *L. helveticus*, *L. iners*, *L. mucosae*, *L. panis*, *L. timonensis*, and *L. ultunensis* were only present in RRC+ patients, contributing to the higher combined relative abundance of *Lactobacillus* species observed in patients with RRC (Fig. 3D *P*-value = 0.006, Mann-Whitney *U* test). Additionally, the abundance of *Candida* species, including *C. albicans, C. dubliens,* and *C. tropicalis* (Fig. 3C), was overall higher although not statistically significant, in the RRC+ group (Fig. 3E *P*-value = 0.12, Mann-Whitney *U* test).

Metagenomic data also confirmed that acid- producing species were more abundant in the dental biofilm of the RRC+ group (Fig. 3G), including *Propionibacterium acidifaciens,* which was several-fold more abundant in patients with RRC. Furthermore, several acid-producing species were only present and enriched in the RRC+ group, including *L. gasseri, Lacticaseibacillus rhamnosus, Limosilactobacillus fermentum, L. vaginalis,* and *Ligilactobacillus salivarius*. Interestingly, we observed a significant positive correlation between *Lactobacillus* and *Candida* species in the dental biofilm of RRC+ patients (Fig. 3F *P*-value = 0.021, *ρ* = 0.47, Spearman’s rank correlation), suggesting that the production of acid metabolites by *Lactobacillus* species could facilitate oral colonization by *Candida* species. An enrichment of health-related commensals was observed in the RRC− group, including *Actinomyces johnsonii*, *Neisseria elongata*, *Cardiobacterium valvarum*, *Gemella morbillorum*, *Eikenella corrodens*, and *Corynebacterium durum* (Fig. 3G).

### Altered potential of energy-related pathways on commensal bacteria

**Fig 4:**
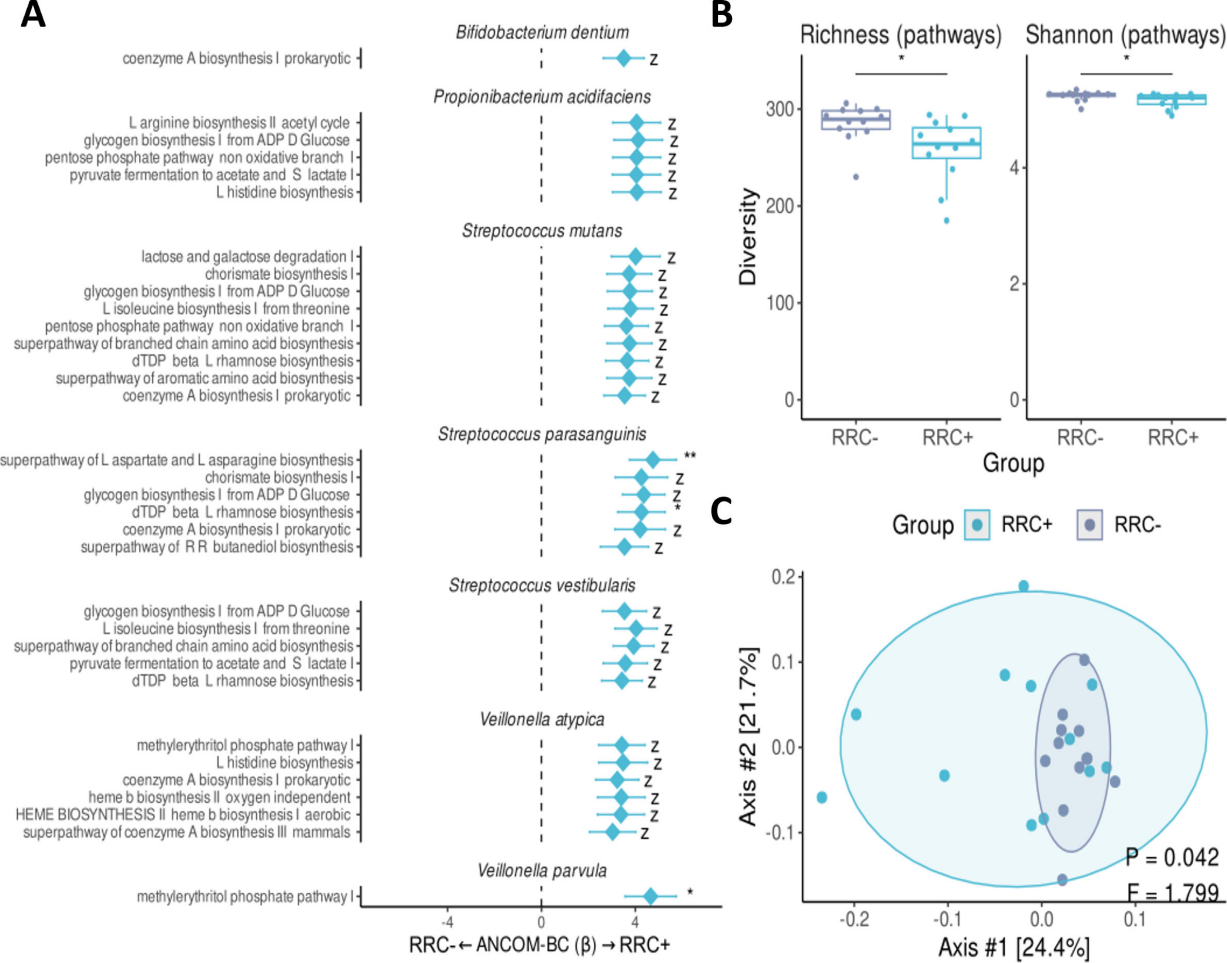
Commensal species of *Streptococcus* and *Veillonella* have a higher potential for expressing energy-metabolic potential in the dental biofilm of the RRC+ group. Enrichment of energy-related pathways for each species (**A**), the number of pathways were measured by richness (**A**) and Shannon’s index (**B**). The metabolic pathways composition of each sample was represented by PCoA (**C**). Differential compositions are presented for metabolic pathways based on the MetaCyc database. Each box represents the median and percentiles (25th and 75th), with lines extending to the extreme (at most 1.5 times the size of the box). Only taxa with significantly altered enrichment are shown. The asterisk represents statistical significance: *, *P*-value < 0.05; **, *P*-value < 0.01; ***, *P*-value < 0.001; *z*, structural zero.

Finally, we evaluated if the differences in the dental biofilm bacterial composition observed between RRC+ and RRC− patients affected the metabolic potential of the microbial community. We found an enrichment of genes linked to energy-related pathways associated with the synthesis of amino acids and sugars in the RRC+ compared to the RRC− group (Fig. 4A). Although pathways from the abundant *P. acidifaciens* contributed to this enrichment, it was driven mainly by genes from commensals species present in both groups, such as *S. parasanguinis, S. vestibularis, S. mutan*s, *Veillonella parvula*, and *V. atypica*. (Fig. 4A). We also observed lower richness (*P*-value = 0.015, Mann-Whitney *U* test) and diversity (*P*-value = 0.028, Mann-Whitney *U* test) of metabolic pathways in the RRC+ group, which aligns with the lower microbial diversity previously observed in this group (Fig. 4B). Interestingly, the composition of metabolic pathways in the RCC+ group was not only different (Fig. 4C, PERMANOVA, *P*-value = 0.042, *F* = 1.799) but also more variable compared to the RRC− group (*P*-value = 0.047, *F* = 4.011, PERMDISP), which might be metabolic pathway-level evidence of an Anna Karenina effect in the perturbed RRC+ ecosystem (43).

## DISCUSSION

RRC significantly impairs the stomatognathic system. Despite its high incidence, the pathophysiology of RRC remains unclear, and preventive guidelines are primarily derived from knowledge of non-irradiated dental caries. This knowledge gap combined with non-targetable risk factors and imprecise guidelines jeopardizes the oral health of HNC survivors. This study investigated the microbial differences between patients who develop RRC post-radiotherapy and those who do not by collecting samples from three oral sites of HNC patients exposed to radiotherapy. We also compared demineralized dental tissue from RRC and CC. Additionally, we performed for the first time metagenomic sequencing on dental biofilm samples from irradiated HNC patients, unveiling species-level insights to better understand RRC etiology. Finally, we collected extensive clinical data on oral health status, radiotherapy planning, and long-term supportive care follow-up to complement the microbial data.

In line with previous work (9, 44), our study found that poor pretreatment oral status was associated with RRC incidence. In our study population, fewer teeth and a higher proportion of rehabilitated teeth before radiotherapy were risk factors for RRC development. Other clinical parameters, such as gingival recession and periodontal pocket depth, have been associated with RRC development (9, 44) but were not evaluated in the present work. Our study did not identify a direct relationship between radiation dose and the onset of RRC. Nevertheless, it is widely recognized that radiation can have an adverse effect on tooth structure, and thermal contractions induced by radiation therapy may promote bacterial infiltration. Subsequent studies should explore alterations in the oral microbiome in conjunction with the evaluation of radiation-induced changes in the morphological, mechanical, and chemical properties of permanent teeth.

Similarly, our analysis did not uncover a direct association between the radiation dose to major salivary glands, the incidence of xerostomia, and the development of RRC. This finding aligns with prior research that also found no significant association between salivary flow and RRC incidence (21, 44). In addition, we found no associations between the radiation dose administered to the parotid gland and changes in the oral microbiome among irradiated patients. Salivary gland damage directly impacts the patient’s quality of life and deserves proper clinical attention to restore oral health (45–47). Although salivary gland hypofunction has long been hypothesized as the sole cause of RRC, no longitudinal studies have so far quantitatively correlated salivary gland irradiation dose with bacterial diversity decline or shifts in microbial composition in patients with RRC. Some studies have reported stable microbial diversity during radiotherapy (17, 18, 48) although ulcerated oral mucositis can disrupt this stability (49, 50). It is worth noting that xerostomia data in our study were collected retrospectively, and forthcoming studies should consider the assessment of xerostomia at the time of oral sample collection, while also including an analysis of changes in the physicochemical properties of the saliva.

The microbiota profile of RRC tissue revealed lower diversity, with enrichment of the *Lactobacillus* genus (51). This finding may explain the aggressive nature of RRC compared to conventional caries. *Lactobacillus* species, lacking specific adhesins, are well-adapted to colonize retentive niches such as dental cavities, where their high acid production capacity confers an ecological advantage. In addition, the enrichment of other lactic acid bacteria, including *Limosilactobacillus*, may enhance the advantage through hydrogen peroxide (H₂O₂) production (52), thereby creating a hostile environment for non-aciduric and competing microbes (53, 54). Further investigations into the specific roles of these microorganisms in RRC pathogenesis are warranted.

Beyond the distinct microbial profile of RRC tissue, we examined the impact of RRC on other oral microbial communities. Our findings revealed that RRC+ patients displayed lower bacterial diversity in the dental biofilm and gingival crevicular fluid. We also found that bacterial composition in RRC+ patients differed significantly from RRC− patients at all oral sites and that the presence of RRC in the oral cavity led to oral dysbiosis and increased similarity in microbiota among the three oral sites. Despite the decrease in microbial diversity in the gingival crevicular fluid of RRC+ patients, we did not observe an overgrowth of periodontal pathogens, and further studies should confirm the lack of increased risk of periodontal disease onset post-radiotherapy. The dental biofilm showed the most significant differences in bacterial composition between the RRC+ and RRC− groups, with enrichment of *Lactobacillus* and *Limosilactobacillus* in RCC+ patients documented by both 16S rRNA sequencing and shotgun metagenomic sequencing. The increased abundance of *Lactobacillus* in the oral cavity of HNC patients treated with radiotherapy was previously reported in other studies (17, 19, 21). Interestingly, low salivary pH has been linked to an increased incidence of RRC (21, 44), and the observed enrichment of acidogenic bacteria may contribute to low salivary pH, independently of salivary flow, and promote the development of RRC.

Finally, based on shotgun metagenomic sequencing, our findings show that the dental biofilm of patients with RCC contains not only differentially abundant species but also an altered overall metabolic potential of the microbial community. The differences we observed at both the taxonomic and functional levels indicate that the presence of RRC in the oral cavity significantly impacts the dental biofilm, even in teeth that have not yet been affected by carious lesions. By combining taxonomic and microbiome functional potential evidence, we propose that dynamic microbial cooperations are established in the irradiated dental biofilm of RRC+ patients. Specifically, early colonizers, such as *Streptococcus* and *Veillonella*, could exhibit higher expression of energy-related metabolic pathways, promoting the production of pyruvate acid and coenzyme A—necessary for heterolactic and homolactic fermentation (55). The availability of pyruvate for degradation into propionate, lactate, acetate, and butyrate—produced by *Propionibacterium*, *Lactobacillus*, and *Bifidobacterium (42),* respectively—might create an acid-inhospitable microenvironment (56–58), which limits the growth of non-aciduric species but could support the proliferation of acidophilic bacterial and fungal species in the dental biofilm. In this context, the unique presence of *Candida* species in the RRC+ dental biofilm warrants further exploration to determine if this is due to opportunistic growth, symbiosis with cariogenic pathogens (59) without participation in the disease, or active role in RRC pathophysiology and dental colonization (60). Our data highlight the complexity of the dental biofilm (40, 41, 61–63) and how the dental biofilm microbial composition can be influenced by cancer treatment and toxicities (64). This study has limitations, including its single-center design, the absence of oral hygiene data during long-term follow-up, and the use of a low-resolution microbiota profiling technique (i.e., 16S rRNA amplicon sequencing) for most of the samples. To enhance mechanistic understanding, metabolomics and spatial-based techniques such as CLASI-FISH (62) could provide valuable insights into the microbial architecture and the ecology of irradiated dental biofilm.

### Conclusions

The cariogenic potential of the oral microbiome in healthy subjects has been well-documented in scientific literature. However, no studies have been conducted on the oral microbiome of irradiated patients with RRC. We showed that RRC tissue had lower bacterial diversity and higher abundance of Lactobacillaceae members compared to CC. In addition, RRC+ patients had lower microbiota diversity and significantly distinct microbiota compositions in comparison with RRC− patients across different oral sites. This was particularly noticeable for the dental biofilm of RRC+ patients, which displayed striking alterations in microbiome composition compared to RRC− patients, including enrichment of acidogenic species and altered metabolic potential.

Developing effective guidelines to prevent RRC requires a comprehensive understanding of dental biofilm growth, formation, and microbial metabolites. Our results highlight the critical importance of oral health education and care for individuals with HNC undergoing radiotherapy. We advocate for the prescription of fluoride products to promote the formation of pH-resistant fluorapatite. We also underscore the significance of using antimicrobial strategies, such as chlorhexidine or antimicrobial photo biomodulation, on enamel and dentin before the application of dental materials during the restorative management of RRC. Future longitudinal studies are warranted to assess whether the observed microbial imbalance in RRC patients is a lasting condition or if dental rehabilitation can facilitate the restoration of ecological balance.

## Data Availability

The 16S rRNA amplicon sequencing data and the shotgun metagenomic sequencing data are available in NCBI-SRA under the accession code PRJNA1093152. Metagenome-assembled genomes generated from the shotgun metagenomic data are also available (65).

## SUPPLEMENTAL MATERIAL

Data S1 msphere.00257-25-s0001.csv Shotgun metagenomic sequencing pre-processing statistics.

Data S2 msphere.00257-25-s0002.csv MaAsLin2 analysis: correlation between genus and irradiation dose in salivary glands and dental arches.

Supplemental Material msphere.00257-25-s0003.pdf Supplemental figures and tables; Data S1 and S2 captions.

ASM does not own the copyrights to Supplemental Material that may be linked to, or accessed through, an article. The authors have granted ASM a non-exclusive, world-wide license to publish the Supplemental Material files. Please contact the corresponding author directly for reuse.
