# Supplementary material for: Dental biofilm serves as an ecological reservoir of acidogenic pathobionts in head and neck cancer patients with radiotherapy-related caries
Source: mSphere. 2025 Jun 30;10(7):e00257-25. doi: 10.1128/msphere.00257-25 (PMC12306161; doi:10.1128/msphere.00257-25)
Supplement: Supplemental Material — Supplemental figures and tables; Data S1 and S2 captions. [file msphere.00257-25-s0003.pdf]

Supplemental materials

Dental biofilm serves as an ecological reservoir of acidogenic pathobionts in head and neck cancer patients with radiotherapy-related caries.

AUTHORS: Julia S. Bruno[a], Vitor Heidrich[b], Felipe C.F. Restini[c], Tatiana M.M.T. Alves[c], Wanessa Miranda-Silva[a], Franciele H. Knebel[a], Elisangela M. Cóser[a], Lilian T. Inoue[a], Paula F. Asprino[a], Anamaria A. Camargo[a#]*, Eduardo R. Fregnani[a#]

AFFILIATIONS
[a] Molecular Oncology Center, Hospital Sírio-Libanês,01308-060, São Paulo, Brazil
[b] CIBIO, University of Trento, 38123, Trento, Italy
[c] Radiotherapy Department, Hospital Sírio-Libanês, 01308-050, São Paulo Brazil

[#]These authors contributed equally.
*Corresponding author.

FILE DESCRIPTION

Figures

Supplementary Fig. S1. Sample collection event in correlation with the follow-up period of the study, in months.
Supplementary Fig. S2. Shotgun metagenomic data comparing RRC- and RRC+ groups.

Tables

Supplementary Table S1. General clinical characteristics of the CC vs RRC- vs RRC+ groups.
Supplementary Table S2. Oncological data of the study population.

Data

Supplementary Data S1: Shotgun metagenomic sequencing pre-processing statistics (separated file .csv)
Supplementary Data S2: MaAsLin2 analysis – correlation between genus and irradiation dose in salivary glands and dental arches. (separated file .csv)

FIGURES

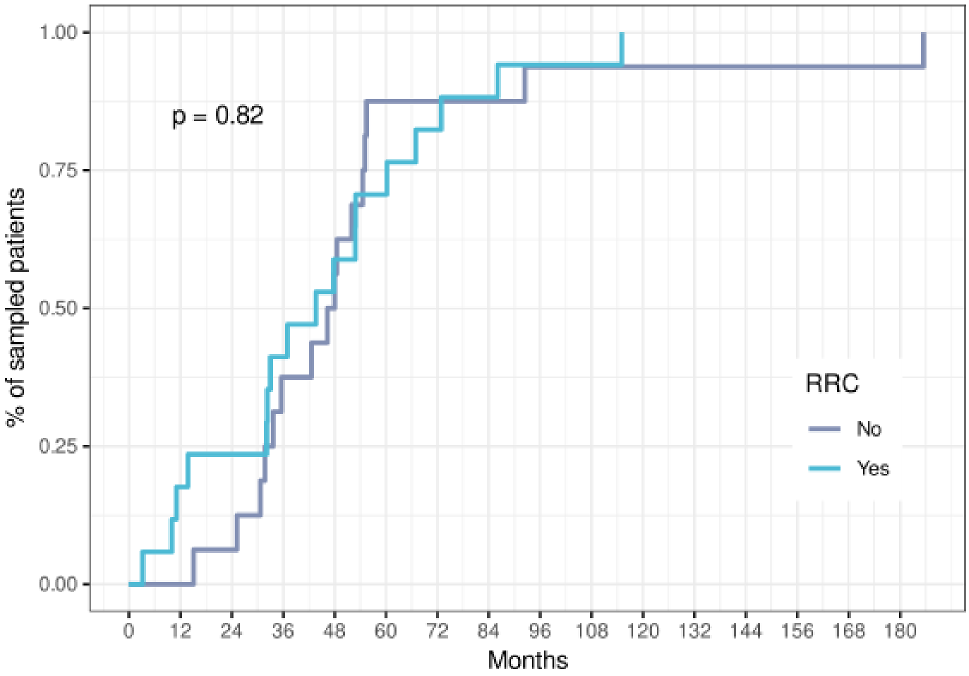

Supplementary Fig. S1. Sample collection event in correlation with the follow-up period of the study, in months. The purple line indicates the sample collection of the RRC- group, and the blue line indicates the RRC diagnosis and samples collection of the RRC+ group. The last radiotherapy session is considered the mark zero.

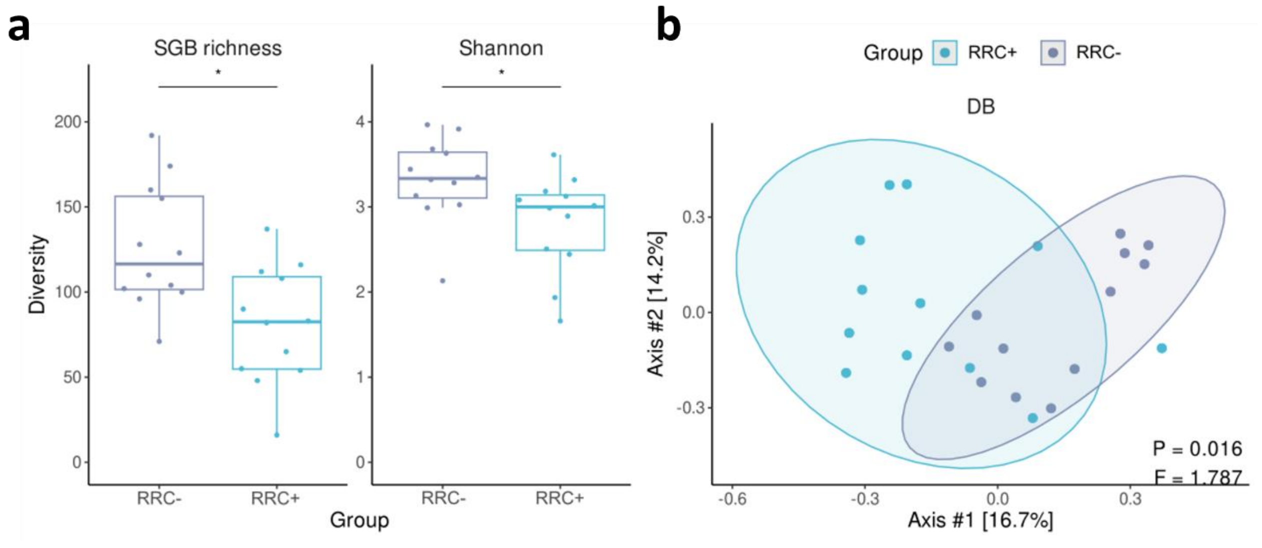

Supplementary Fig. S2. Shotgun metagenomic data comparing RRC- and RRC+ groups. The RRC+ group had lower diversity on SGB richness and Shannon's diversity index (a) and based on the Bray-Curtis metric, RRC+ group presented samples with distinct compositions (b), represented by PCoA. Each box represents the median and percentiles (25th and 75th) with lines extending to the extreme (at most 1.5 times the size of the box). The asterisk represents statistical significance: *, p-value < 0.05.

TABLES

Supplementary Table S1. General clinical characteristics of the CC vs RRC- vs RRC+ groups

Clinical data (n=49)	Study population			**p-value, test**
	**CC** (n=16)	**RRC-** (n=16)	**RRC+** (n=17)	
**Age - years (mean ± s.d.)**	56.8 ± 18.4	54.9 ± 11.5	68.5 ± 10.5	**0.0144**, a
**Gender - male, no. (%)**	9 (56.25%)	10 (62.50%)	12 (70.58%)	0.6924, b
**Comorbidities - no. (%)**				
No history of systemic disease	12 (75%)	12 (75%)	12 (70.58%)	0.9461, b
Systemic Arterial Hypertension	0	3 (18.75%)	4 (23.52%)	0.1658, c
Controlled Diabetes Mellitus	4 (25%)	3 (18.75%)	1 (5.88%)	0.3126, c
**Tobacco consumption - no. (%)**				
No usage history†	14 (87.5%)	10 (62.5%)	11 (64.70%)	0.2202, b
Smoker ‡	2 (12.5%)	2 (12.5%)	2 (11.76%)	0.4805, c
Former smoker‡‡	0	4 (25%)	2 (11.76%)	

†: Defined as those who never consumed more than 5 packs/100 cigarettes in their lifetime; ‡: Smoker: Defined as those who have already smoked 100 cigarettes in their lifetime and maintain continuous daily use. ‡‡Former smokers are defined as those who have already used more than 100 cigarettes but do not smoke for a period ≥ 2 years at the time of the sample collection.

a: One-way ANOVA;b: Chi-square;c: Fisher-Freeman-Halton Test.

Supplementary Table S2. Oncological data of the study population

	Study Population		p-value, test
	**RRC-** (n=16)	**RRC+** (n=17)	
**Histological type - no. (%)**			
Squamous Cell Carcinoma	15 (88.23%)	17 (100%)	0.4848, e
Other	1 (6.25%)	0	
**Primary tumour site - no. (%)**			
Oral Cavity	7 (43.75%)	7 (41.17%)	0.8729, c
Oropharynx	6 (37.5%)	5 (29.41%)	
Nasopharynx	2 (12.5%)	3 (17.64%)	
Larynx	1 (6.25%)	2 (11.76%)	
**Tumor - no. (%)**			
Tx	1 (6.25%)	2 (11.76%)	0.4662,c
T1-T2	10 (62.5%)	7 (41.17%)	
T3-T4	5 (31.25%)	8 (40.05%)	
**Nodes - no. (%)**			
Nx	1 (6.25%)	2 (11.76%)	0.5935,c
N0-N2a	11 (68.75%)	12 (70.58%)	
N2b-N3	4 (25%)	2 (11.76%)	
**Metastasis - no. (%)**			
M0	15 (93.75%)	17 (100%)	0.4848,e
M1	1 (6.25%)	0	
**Surgery - no. (%)**			
Primary site	8 (50%)	8 (40.05%)	0.8658, b
**Chemotherapy - no. (%)**			
Cisplatin	3 (18.75%)	8 (40.05%)	0.3618, e
Combined Chemotherapy ✢	5 (31.25%)	4 (23.52%)	

**Oral toxicities - no. (%)**			
Oral Mucositis †			
Grade 1-2	10 (62.5%)	9 (52.94%)	0.4905, e
Grade 3-4	5 (31.25%)	8 (47.05%)	
Xerostomia ‡			
Grade 1-2	13 (81.25%)	10 (58.825)	0.2587, e
Grade 3	3 (18.75%)	7 (41.17%)	

✢: Combined Chemotherapy: Cisplatin+5FU+Paclitaxel or Cisplatin+Paclitaxel or Cisplatin + 5-FU; †: Toxicity graded during RT; ‡: Toxicity graded at sample collection. Tests: b: Chi-square; c: Fisher-Freeman-Halton Test; e: Fisher's exact test.
